# Dynamic fingerprinting of sub-cellular nanostructures by image mean square displacement analysis

**DOI:** 10.1038/s41598-017-13865-4

**Published:** 2017-11-01

**Authors:** Luca Digiacomo, Francesca D’Autilia, William Durso, Paolo Maria Tentori, Giulio Caracciolo, Francesco Cardarelli

**Affiliations:** 1 Department of Molecular Medicine, “La Sapienza” University of Rome, Roma, Italy; 2 Center for Nanotechnology Innovation@NEST, Istituto Italiano di Tecnologia, Pisa, Italy; 3 NEST - Scuola Normale Superiore, Istituto Nanoscienze - CNR (CNR-NANO), Pisa, Italy

## Abstract

Here we provide demonstration that image mean square displacement (*i*MSD) analysis is a fast and robust platform to address living matter dynamic organization at the level of sub-cellular nanostructures (e.g. endocytic vesicles, early/late endosomes, lysosomes), with no a-priori knowledge of the system, and no need to extract single trajectories. From each *i*MSD, a unique triplet of average parameters (namely: diffusivity, anomalous coefficient, size) are extracted and represented in a 3D parametric space, where clustering of single-cell points readily defines the structure “*dynamic fingerprint*”, at the whole-cell-population level. We demonstrate that different sub-cellular structures segregate into separate regions of the parametric space. The potency of this approach is further proved through application to two exemplary, still controversial, cases: *i*) the intracellular trafficking of lysosomes, comprising both free diffusion and directed motion along cytoskeletal components, and *ii*) the evolving dynamic properties of macropinosomes, passing from early to late stages of intracellular trafficking. We strongly believe this strategy may represent a flexible, multiplexed platform to address the dynamic properties of living matter at the sub-cellular level, both in the physiological and pathological state.

## Introduction

A distinguishing feature of eukaryotic cells at the sub-cellular spatial scale is that of being organized into membrane-enclosed, sub-micrometric, and dynamic organelles or compartments, such as endocytic/secretory vesicles, early-late endosomes, lysosomes, mitochondria, etc.^1^. As testified, among others, by the 2013 Nobel Prizes in Physiology or Medicine^2^, such structures are pivotal in determining how cells shuttle proteins and other biomolecules from one location to another, thus emerging as a legitimate platform for cell signaling regulation^3^. The overall picture is that a tight regulation of their size/morphology and trafficking properties determines how cells are able to comply with internal or external stimuli both in the physiological and in the pathological conditions^4–6^. In spite of the recent advances in live-cell imaging technologies (e.g. development of organelle-specific markers, optimization of labeling procedures, and availability of highly sensitive optical microscopes^7–9^), observing subcellular structures at high spatial and temporal resolution, and in living cells, is still a challenging task. Concerning the analytical tools used, historically, Single Particle Tracking (SPT) is the technique of choice for sub-cellular structures^10–15^. Although it provides an extremely high and detailed amount of information on the system, however, extraction and processing of single trajectories renders SPT an inherently time-consuming approach. In this regard, our groups recently proposed a fluorescence-based spatiotemporal fluctuation analysis method that makes it possible to extract the average dynamic behavior of diffusing objects directly from standard imaging, in the form of a mean square displacement (MSD) versus time-delay plot (hereafter referred to as image-derived MSD, or *i*MSD)^16–19^. The *i*MSD approach does not need any preliminarily assumption/knowledge on the system under study and, more importantly, does not need to extract the single trajectories. Of particular note, the *i*MSD plot simultaneously yields information also on the average size of the diffusing object by its offset for time delays approaching to 0 (property discussed in ref.^16^), thus holding the potential to offer a fast and robust alternative strategy to probe the characteristic structural and dynamic properties of sub-cellular structures under different conditions. Thus far, the potential of the *i*MSD approach was demonstrated in a number of applications on molecules (mostly reviewed in ref.^18^) and on gene-delivery nanoparticles^20^. Here, we show the applicability of this method to one of the natural conditions of living matter at the sub-cellular spatial scale, that of sub-micrometric, dynamic structures or compartments deputed to shuttle molecules around the cell. To this end, we used fluorescently labelled variants of the major structures involved in endocytic processes (namely: caveolae, clathrin-coated vesicles, and macropinosomes) and in the subsequent intracellular processing of internalized cargoes (namely: early endosomes, late endosomes, and lysosomes). For each of these structures, the local diffusivity (D_micro_, hereafter D_m_), the anomalous-diffusion coefficient (α), and the offset parameter (σ_0_^2^) are extracted by fitting the *i*MSD plot and represented in a parametric 3D space. In this way, the whole population of diffusing objects probed for each cell will be represented by a unique triplet of parameters, i.e. by a single point in the 3D plot. Thus, clustering of single-cell experimental points readily depicts what we call the “dynamic fingerprint” of the selected structure at the whole-cell-population level. Worthy of note, by this approach we easily demonstrate that each of the analyzed structures segregates into a separate region of the chosen parametric space, according to the almost unique combination of its characteristic average size and dynamic properties. To show the potency of this approach, we then tackled two exemplary cases. First, we probed the dynamic properties of the lysosome, and found that a super-diffusive mode of motion emerges as a collective behavior at short spatiotemporal scales, while sub-diffusion dominates at longer spatiotemporal scales. Second, we probed the time-evolution of the macropinosomes fingerprint during the lifespan of their trafficking, from early appearance at the plasma membrane to late stages of intracellular processing. To our knowledge, this is the first time that collective properties (and their time evolution) are quantitatively probed for these organelles in living cells. We believe that such a simultaneous, fast, and robust access to average structural and dynamic parameters may become a useful quantitative tool to address living matter organization and function at the sub-cellular spatial scale.

## Results and Discussion

### From the *i*MSD analysis to the dynamic fingerprint of intracellular structures

**Figure 1:**
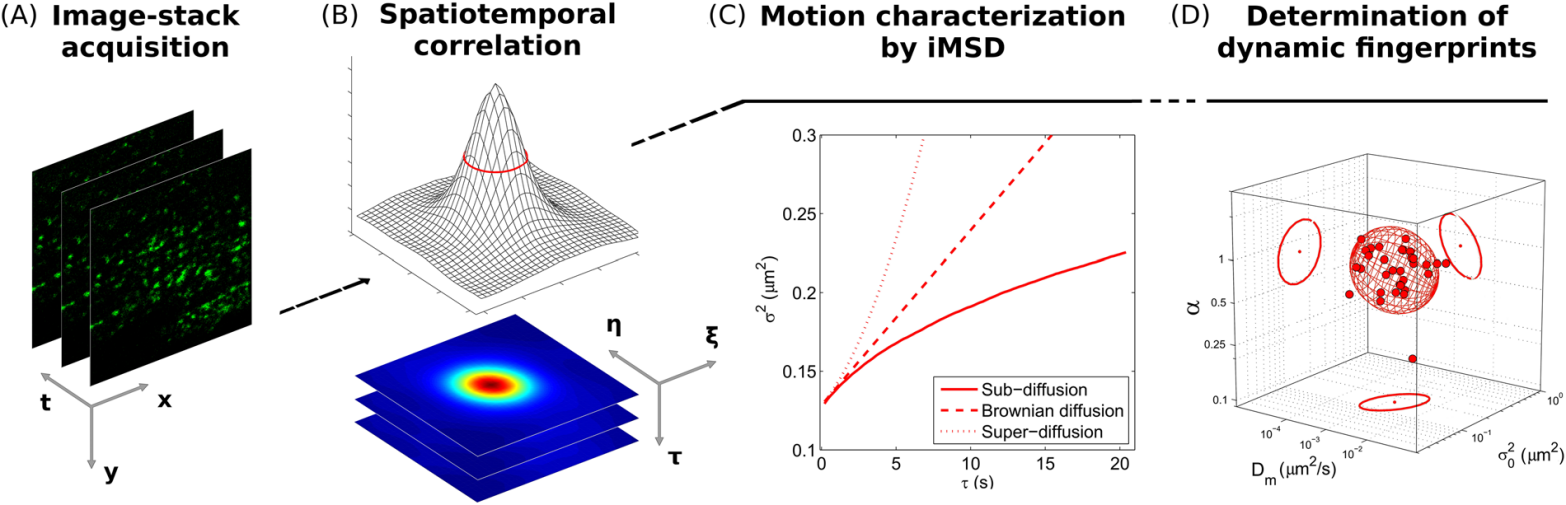
Schematic representation of the *i*MSD-based dynamic fingerprint analysis. (**A**) A stack of images of fluorescently-labelled intracellular structures is acquired by time-lapse confocal microscopy. (**B**) Spatiotemporal correlation function is derived from image analysis by the *i*MSD algorithm (see Materials and Methods for equations). (**C**) Gaussian fitting of correlation functions allows to extract the *i*MSD plot, which in turn depicts the average diffusion law of the structure of interest (exemplary cases are reported: super-diffusion, dotted red line; isotropic diffusion, dashed red line; sub-diffusion, solid red line). (**D**) Three relevant parameters are chosen from the fitting equation (see Materials and Methods) to quantitatively describe the average dynamic properties of the structure of interest, namely: the short-range diffusion coefficient (D_m_), the anomalous diffusion coefficient (α), and the y-axis intercept of the *i*MSD plot, indicating the average size of the diffusing structures. These three parameters are organized in a 3D plot, used to identify the ‘dynamic fingerprint’ of the diffusing structure.

The typical scheme of an *i*MSD experiment is illustrated in Fig. 1 (also discussed in detail in previous reports^16–18,20^), while the typical data-analysis output is reported in Supp. Fig. 1. In brief, the starting point is fast imaging of a given region of interest within the cell (the entire cytoplasm in this case) (Fig. 1A). Then, the spatiotemporal correlation function is calculated comparing acquired images at increasing time delays, for example each 2, 3, n repetitions (Fig. 1B). The width of the peak of the spatial autocorrelation function increases at increasing time delays as a function of the movement of the object of interest, independently of its particular mode of motion. By fitting the spatiotemporal correlation function, the average *i*MSD can be extracted (Fig. 1C). Three main *i*MSD results might be expected, that is: isotropic diffusion (dashed red line), super-diffusion (dotted red line), or sub-diffusion/confined motion (solid red line) (Fig. 1C). Thus, through fitting procedures of the spatiotemporal correlation function, we determined the kind of motion and measured all the relevant dynamic parameters. Among them, we especially focused on anomalous diffusion exponent α, short-term diffusion coefficient D_m_ and intercept value σ_0_^2^. Of note, the square root of this latter parameter is proportional to the average size of the diffusing objects (property discussed in detail in ref.^16^). In this way, for each of the investigated samples, the measured values can be represented in a 3D parameter-space, within which the results of a specific image-stack correspond uniquely to a data point (Fig. 1D). In turn, a set of image-stacks exploring the dynamics of a specific sub-cellular structure defines a multivariate 3D distribution, with peculiar location and orientation in the parameter-space. We quantified these geometrical features by diagonalizing the covariance matrix of the distribution, then we defined the resulting ellipsoid according to the obtained eigenvalues and eigenvectors. In this way, the space within the ellipsoidal surface can be regarded as the analogue of the confidence interval [mean − SD, mean + SD] for 1-dimensional distributions. Thus, this procedure provides a graphical visualization of extension, position and rotation of each distribution, which here is referred to as the *dynamic fingerprint* of the corresponding sub-cellular structure.

### The dynamic fingerprint of exemplary sub-cellular nanostructures: endocytic vesicles and the endosome-lysosome pathway

**Figure 2:**
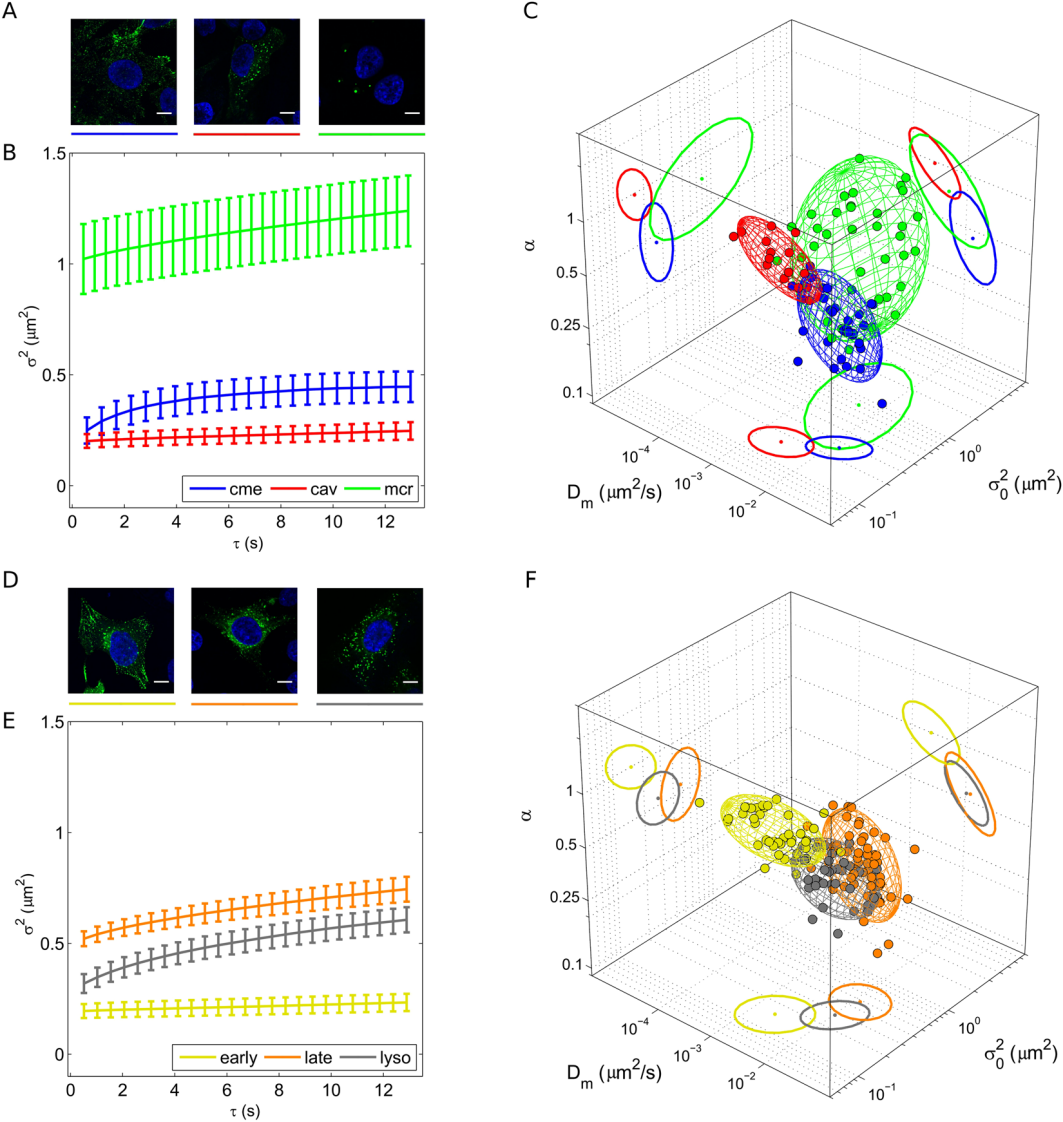
Dynamic fingerprint of exemplary intracellular nanostructures. (**A**) Representative confocal images of clathrin-coated vesicles, macropinosomes, and caveolae, respectively (green signals). The blue signal indicates the nucleus. Scale bar: 10 µm. (**B**) Representative *i*MSD plots of the three structures with error bars (clathrin-coated vesicles in blue, macropinosomes in green, and caveolae in red). (**C**) 3D plot of extracted parameters shows the differences in the dynamic (short-range diffusivity and anomalous coefficient) and structural (size) properties of the selected structures. (**D**) Representative confocal images of early endosomes, late endosomes, and lysosomes, respectively (green signals). Scale bar: 10 µm. (**E**) Representative *i*MSD plots of the three structures with error bars (early endosomes in dark yellow, late endosomes in orange, and lysosomes in grey). (**F**) 3D plot of extracted parameters shows the differences in the dynamic and structural properties of the selected structures.

Figure 2A and B show live-cell confocal images (for labelling procedures see Supplementary Materials and Methods) and representative *i*MSD traces of three well known subcellular structures involved in endocytic processes in HeLa cells, namely caveolae (red curve), clathrin-coated vesicles (blue curve) and early macropinosomes (green curve). The definition ‘early’ is here used to distinguish measurements performed on macropinosomes at early stages of trafficking (i.e. within 80 minutes after administration) from those performed at an intermediate stage (i.e. from 80 to 120 minutes after administration) and from those performed at late stages (i.e. after more than 120 minutes from administration). These latter cases are discussed later in the text. The *i*MSD traces, already by visual inspection, reveal clear differences in the characteristic structural and dynamic properties of the selected structures. More quantitatively, the extracted parameters (D_m_, α, and σ_0_^2^) are combined together, as described above, to highlight the characteristic dynamic fingerprint of each structure (Fig. 2C) (distributions of D_m_, α, and σ_0_^2^ are reported in Supp. Fig. 2). A few aspects are worthy of mention. First, the three pathways are clearly distinguishable one from the other in terms of the average characteristic size of the structures involved. Although we cannot directly compare our data with previous electron-microscopy-based analyses on these organelles (e.g.^21–23^), the retrieved σ_0_^2^ values nicely match the expectations, with macropinosomes being, on the average, larger than clathrin-coated vesicles and caveolae (Fig. 2C, see also Table 1). In addition, from a dynamic point of view, clathrin-mediated endocytosis appear to involve structures substantially more mobile, in terms of local diffusivity (D_m_ = [16.2 ± 9.9] × 10^−3^ µm^2^/s), as compared to both caveolae ([3.1 ± 1.8] × 10^−3^ µm^2^/s) and macropinosomes ([8.3 ± 9.0] × 10^−3^ µm^2^/s). This result is not surprising, as clathrin-coated vesicles are known to readily detach from the plasma membrane (in a few seconds^24^) to become free to move across the cytoplasm. By contrast, caveolae are characterized by quite long residency times at the plasma membrane, and slow intracellular trafficking^25^. Similarly, macropinosomes at an early stage of formation are large, membrane-bound structures, whose detaching and subsequent trafficking necessitates coordinated cellular processes to be executed, such as actin polymerization, ruffle closure, etc.^26^. Interestingly, however, the combination of such differences in local mobility of these structures with their respective average anomalous coefficient (α = 0.48 ± 0.17 for clathrin-mediated endocytosis, and α = 1.00 ± 0.22 and 0.79 ± 0.27 for caveolae and macropinosomes, respectively) produce the effect that they become quite similar if compared on the basis of their long-range diffusivity (D_M_, see Materials and Methods; values are reported in Table 1), i.e. they are able to explore the intracellular environment to a very similar extent, at large spatial scales. At this point, we sought to determine the dynamic fingerprint of one of the main intracellular pathways devoted to the processing of internalized cargoes, that is: early endosomes, late endosomes and lysosomes, also known as the ‘endosomal-lysosomal system’^27^ (representative images of their fluorescently labelled variants and average *i*MSD curves are shown in Fig. 2D,E). The extracted parameters (distributions of D_m_, α, and σ_0_^2^ are reported in Supp. Fig. 3) depict a scenario in which the transition from early to late endosomes entails the involvement of larger (shift in σ_0_^2^, from 0.16 ± 0.08 µm^2^ to 0.50 ± 0.15 µm^2^) and more mobile (shift in local diffusivity, D_m_, from [3.0 ± 2.4] × 10^−3^ µm^2^/s to [15.4 ± 10.6] × 10^−3^ µm^2^/s) structures (see Fig. 2E,F and Table 1). The increase in size is not surprising, as late endosomes are known to have the morphological characteristics of multivesicular bodies^28^. On the other hand, the increased diffusivity of late endosomes can be linked to their preferential localization in the center of cell cytoplasm, as compared to early endosomes, which are mainly membrane-located organelles^29^. Also, the (apparent, as explained above) contradiction of the Stokes-Einstein relation at short spatial scales is compensated, at larger spatial scales, by the differential contribution of the anomalous coefficient α (1.02 ± 0.20 for early endosomes and 0.58 ± 0.16 for late endosomes), so that the long-range dynamic behavior of these structures is quite similar (D_M_ values in Table 1). Finally, transition from late endosomes to lysosomes entails an expected decrease of the characteristic average size of the structures, while no significant changes in local diffusivity are observed (Table 1).

**Table 1:** Parameters extracted from the *i*MSD fitting. Values are reported as Mean ± Standard Deviation. The number of cells analyzed for each sample is reported in the first column, within brackets.

	α	σ_0_^2^ (μm^2^)	D_m_ (×10^−3^ μm^2^/s)	D_M_ (×10^−3^ μm^2^/s)
cme (33)	0.48 ± 0.17	0.27 ± 0.06	16.2 ± 9.9	2.8 ± 1.3
cav (15)	1.00 ± 0.22	0.17 ± 0.04	3.1 ± 1.8	1.2 ± 0.5
*e*-mcr (23)	0.79 ± 0.27	1.13 ± 0.95	8.3 ± 9.0	2.5 ± 2.2
*i-*mcr (38)	0.60 ± 0.38	0.52 ± 0.28	13.7 ± 19.9	2.2 ± 2.7
*l*-mcr (25)	0.39 ± 0.21	0.37 ± 0.16	5.8 ± 4.7	0.4 ± 0.3
*e*-endo (41)	1.02 ± 0.20	0.16 ± 0.08	3.0 ± 2.4	1.1 ± 0.6
*l*-endo (58)	0.58 ± 0.16	0.50 ± 0.15	15.4 ± 10.6	3.1 ± 1.8
lyso (60)	0.56 ± 0.12	0.29 ± 0.10	12.3 ± 7.0	2.7 ± 1.0
lyso_*fast* (26)	1.24 ± 0.13	0.32 ± 0.08	11.9 ± 7.3	10.4 ± 7.6
lyso_*medium* (26)	0.55 ± 0.13	0.33 ± 0.10	13.0 ± 6.7	2.6 ± 1.5
lyso_*slow* (26)	0.33 ± 0.10	0.39 ± 0.10	10.4 ± 3.7	1.2 ± 0.5
lyso *fast* + NCZ (7)	0.81 ± 0.33	0.39 ± 0.08	3.3 ± 1.6	1.6 ± 1.0

### The role of the time window: super-diffusive or sub-diffusive lysosomes?

**Figure 3:**
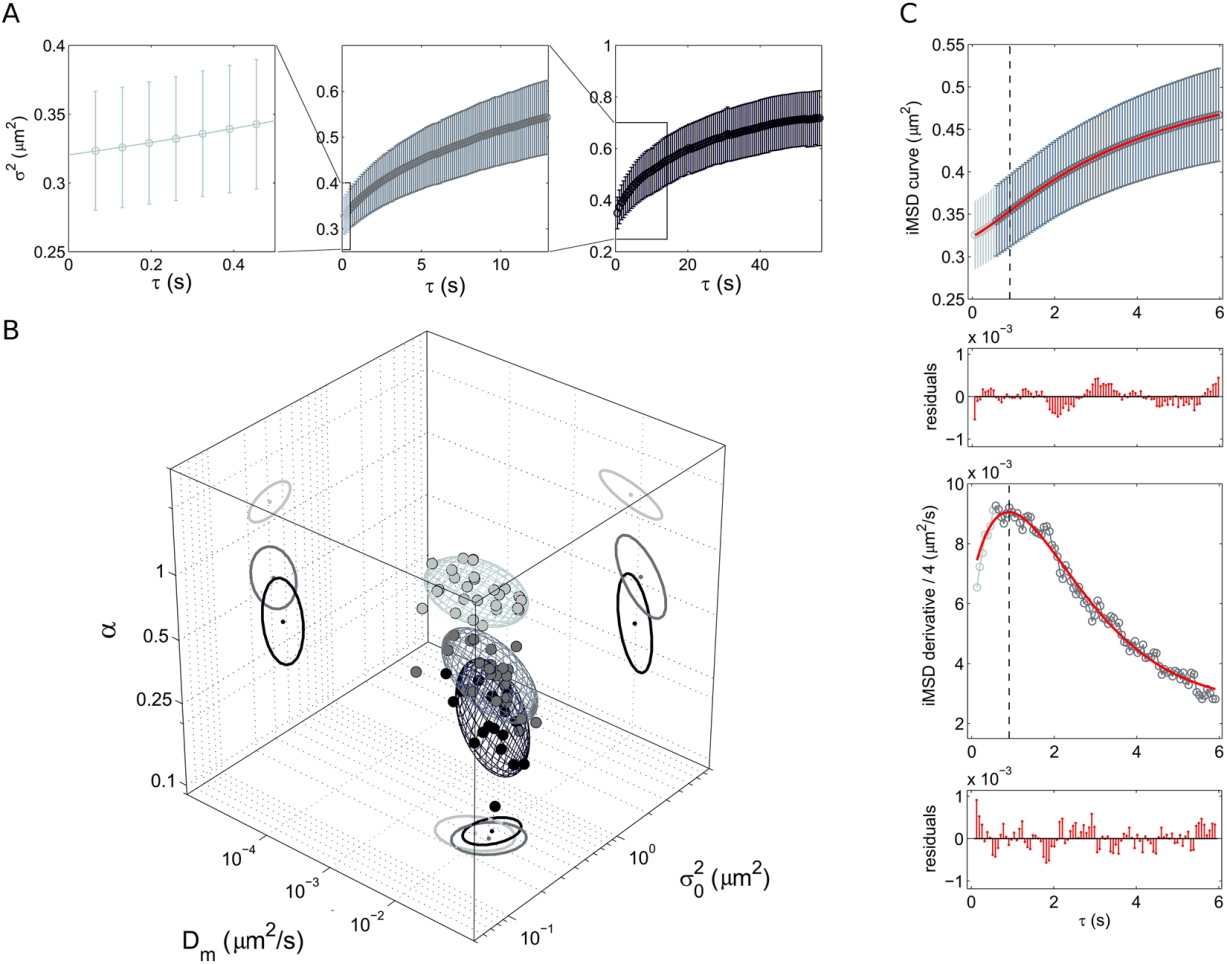
Dynamic fingerprint of the lysosome at different spatiotemporal scales. (**A**) Exemplary *i*MSD plots of the lysosome at three different temporal scales: short, 0–0.5 seconds (left, light grey); intermediate, 0–12 seconds (middle, grey); long, 0–60 seconds (right, dark grey). (**B**) 3D plot of the dynamic fingerprint of the lysosome at the three different temporal scales. (**C**) The first 6 seconds of the *i*MSD plot are reported here (upper panel) to show the goodness of the fit to the global model (red line and residuals, Eq. 6 in Materials and Methods). The derivative of the *i*MSD plot shows the trend of the measured diffusivity (lower panel).

Up to now we compared the different intracellular structures on the common time window of approximately 10 seconds. A fully expected property of the proposed analysis is that the overall shape of the resulting *i*MSD must strictly depend on the timescale considered. A limit case on a very large timescale, for instance, is the total confinement due to the plasma membrane impenetrable boundary, irrespective of the chosen intracellular structure. On the other end, on a very short timescale, one may expect to grab the local mode of motion of the structure. To show how this applies to a real case, we show here an application to the lysosome. Technically, we performed three consecutive measurements on the same cell, at three different timescales, namely: *i*) a short timescale (0–0.5 seconds), *ii*) an intermediate timescale, coincident with that used for all the structures (0–12 seconds), and *iii*) a long timescale (0–60 seconds) (Fig. 3A). At first glance, the *i*MSD analysis reveals three very different dynamic fingerprints of the lysosome (distributions of D_m_, α, and σ_0_^2^ are reported in Supp. Fig. 4). In particular, on the shortest timescale, the *i*MSD plot clearly shows the emergence of an average super-diffusive behavior of lysosomes (α = 1.24 ± 0.13) (Fig. 3). Please consider that this corresponds to active transport of lysosomes along different (random) directions (with a measured speed of about 0.12 μm/s), as no overall movement of the peak of the spatiotemporal correlation function (i.e. concerted movement of the lysosomes in one preferential direction) was detected, see example in Supp. Fig. 1, panel D). Of note, this result speaks in favor of lysosome possible movement along cytoskeleton components (possibly microtubules, based on previous evidences^30,31^). As a control for this, we performed an experiment in presence of 10 µM Nocodazole (see Materials and Methods for more details) to induce selective microtubule depolimerization. As expected, under these conditions, lysosome super-diffusive behavior vanishes in favor of a slightly sub-diffusive local motion (α = 0.81 ± 0.33) and a sensibly reduced local diffusivity (D_m_ = [3.3 ± 1.6] × 10^−3^ µm^2^/s) (Table 1, 3D plot in Supp. Fig. 5). Of note, the super-diffusive behavior vanishes also if we extend the timescale of the acquisition. At an intermediate timescale (the same reported in Fig. 2F) the average dynamics of lysosomes is characterized by α coefficient below 1, indication of a sub-diffusive behavior that can be readily ascribed to the effect of the complex intracellular environment on lysosome movement. This effect is much more evident if the timescale of the acquisition is further extended to 60 seconds (α = 0.33 ± 0.10) (Fig. 3B and Table 1). The detected super-diffusive and sub-diffusive trends at different timescales can be described by a unique model, which quantifies the time extent within which the super-diffusion is dominant, as well as all the other involved dynamic parameters. This description is expressed by Eq. 6 and overcomes the apparent incoherence of the measured α-values associated to the same sub-cellular structure. In this regard, Fig. 3C shows a representative experimental curve and the corresponding fitting curve (red) that has been computed according to Eq. 6. The model is in good agreement with the experimental trend and the transition from super- to sub-diffusion is even more noticeable in the derivative plot, which shows an almost linear increase in the particle mobility at short timescale (super-diffusion) from an intercept value D_m_, until a maximum is reached and then an exponential fall (sub-diffusion) toward an asymptotic value D_M_. In conclusion, we show that lysosome mobility is a combination of active transport and sub-diffusion. By averaging the behavior of many lysosomes at the same time, we can define directed motion and sub-diffusion as ‘collective’ transport properties for this organelle within the cytoplasm. It is worth noting that these two modes of motion were already proposed based on tracking experiments conducted on single, isolated lysosomes^32,33^. What we can add here by *i*MSD analysis is the average spatiotemporal relationship between these two processes: active transport regulates lysosome movement, on the average, at a short timescale (and small spatial scale), while sub-diffusion plays a major role, on the average, at a longer timescale (and larger spatial scale). In this regard, interestingly, Balint *et al*. recently proposed a model of lysosome motion postulating that the intersections between microtubules are able to impose a significant hindrance to directed motion, thus leading to long pauses in transport or, eventually, switches to diffusion^14^. Our results, in keeping with such model, define the average spatial (and temporal) extent of the contribution of directed motion along microtubules.

### The role of the time window: the ‘evolving’ macropinosome

**Figure 4:**
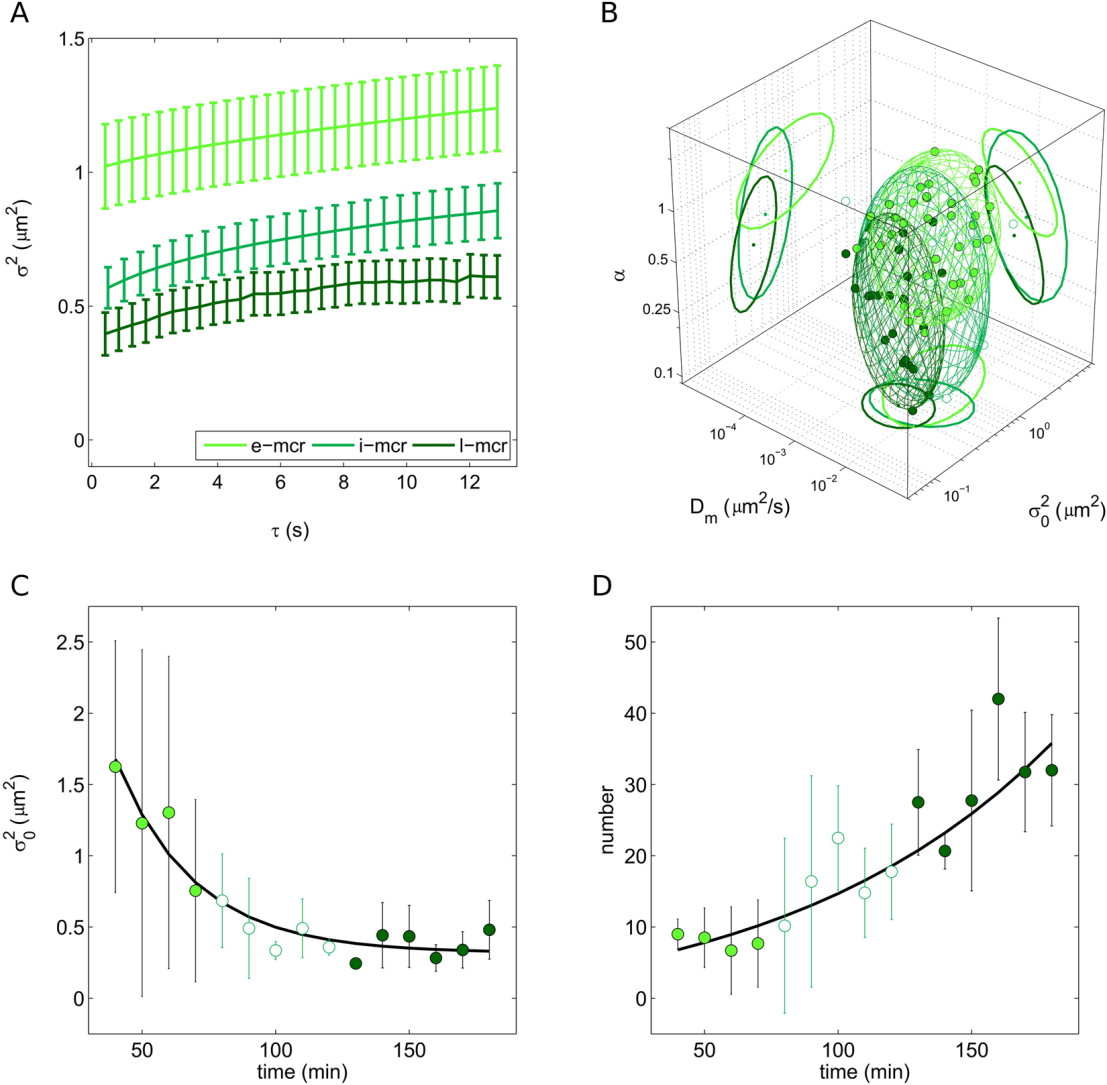
The time evolution of the macropinosome dynamic fingerprint. (**A**) Exemplary *i*MSD plots of macropinosomes at three different stages of trafficking: early (30–70 min), intermediate (80–120 min), and late (>130 min). (**B**) 3D plot showing the time evolution of the dynamic fingerprint of macropinosomes from early (light green) to intermediate (white) and finally late (dark green) stages of trafficking. (**C**) Statistics on the average size (offset, σ^2^) of macropinosomes observed at the different time points. (**D**) Statistics on the average number (N) of macropinosomes observed at the different time points.

As mentioned above, macropinocytosis defines a series of events initiated by extensive plasma membrane reorganization or ruffling to form an external macropinocytic structure that is then enclosed and internalized^21^. Internalized macropinosomes at an early stage of trafficking share many features with phagosomes and both are distinguishable from other forms of endocytic vesicles by their large size (see results in Fig. 2), morphological heterogeneity and lack of coat structures. In general, the paucity of information available on macropinocytosis has hampered efforts to characterize its dynamics and to identify regulatory proteins that are expressed in order to allow it to proceed. What is known from biochemical analyses is that, after internalization, macropinosomes gradually get enriched of regulatory proteins common to other endocytic pathways, in turn suggesting that their identities as unique structures are short-lived^26^. In particular, by using antibodies against known markers of the endosomal pathway in fixed cells, it was shown that macropinosomes progressively develop classical endosomial characteristics before diminishing in size, developing into late endocytic structures (e.g. lysosomes) or eventually losing their identity via membrane retrieval^34,35^. To our knowledge, however, no report thus far addressed this endocytic route (and its temporal evolution) from a dynamic point of view in live cells. Here we address this process by the *i*MSD approach. After 20-min incubation of cells with 70-kDa dextrans, we acquired time series of trafficking macropinosomes at different time points, from 30 minutes up to approximately 3 hours after treatment. For simplicity, we divided the time points into three major groups: early macropinocytosis (30–70 min, already discussed, *e*-mcr in Table 1), intermediate macropinocytosis (80–120 min, *i*-mcr in Table 1), and late macropinocytosis (>120 min, *l*-mcr in Table 1). Nicely, we are able to highlight the gradual change in the structural and dynamic properties of macropinosomes during trafficking (Fig. 4A; distributions of D_m_, α, and σ_0_^2^ are reported in Supp. Fig. 6). While no significant changes in the local diffusivity of macropinosomes occurs during trafficking (Supp. Fig. 7A), both their size and overall mode of motion (α) are substantially modified in time (3D plot of Fig. 4A). In particular, during trafficking, we observe a gradual decrease of the macropinosomes average size (Fig. 4A and B, see also Table 1), and a concomitant increase of the sub-diffusive nature of their motion (Fig. 4A and Supp. Fig. 7B, see also Table 1). As a further analysis, we calculated the number of macropinosomes in each acquisition: results are reported in Fig. 4C and clearly show an increase of N in time. Overall, these observations well depict a scenario in which the macropinosomes originate as large structures at the membrane and then evolve, during intracellular trafficking, by decreasing in size and increasing in number. These results highlight the evolving ‘dynamic nature’ of these organelles that, together with their ‘biochemical nature’, defines their overall functional properties within the cell.

## Conclusions

In this work, we propose the *i*MSD analysis as a promising tool to address the characteristic structural and dynamic properties of sub-cellular nanoscopic compartments in living cells. Starting from a standard stack of images, the *i*MSD algorithm affords simultaneous access to several critical parameters of the diffusing object of interest, such as its average size, diffusivity, and overall mode of motion. Being a fluctuation-based approach, the *i*MSD approach does not rely on single object localization and, as a direct consequence, does not need to extract single-object trajectories, such as in SPT-based experiments. By contrast, it provides rapid and robust quantification over the entire probed area (i.e. over the entire population of diffusing objects). This immediacy of the method is achieved at the expense of recognizing local heterogeneities in the dynamic properties of the structure of interest (e.g. identification of sub-populations and outliers), a prerogative of SPT-based experiments. Overall, the *i*MSD approach must be considered as a tool to get rapid access to information at the whole-cell (and whole-cell-population!) level, thus particularly suited to large-scale screening applications. To show the principle, the tool is used here to build whole-cell-population fingerprints of relevant subcellular structures as well as to probe fingerprint variations in exemplary biological cases. At the same time, the inherent simplicity, robustness, and modest dependence on labelling strategies (and on S/N ratio) of the proposed tool opens the way to straightforward additional applications. For instance, one may consider the *i*MSD-based fingerprint as a tool to recognize and quantitatively describe average alterations in the properties of a particular subcellular structure of interest occurring, for instance, under pathological conditions. A few examples: derailed endocytosis is commonly found in cancer cells^4,5^, altered granule trafficking is found in β-cells exposed to Type-2-Diabetes-mimicking conditions^36^, enlarged lysosomes packed with twisted microtubules are a recognized hallmark of globoid cell leukodystrophy or galactosylceramide lipidosis^37^, abnormalities in the endosomal-lysosomal system are observed in neurodegenerative diseases, especially Alzheimer’s disease (AD)^27^. In this regard, *i*MSD-based fingerprinting can represent a useful methodological platform for rapid screening of different phenotypes, different pharmacological treatments, etc. In addition, we envision the possibility to use the dynamic fingerprint of selected sub-cellular structures as a reference for testing the time evolution of the intracellular trafficking of exogenously added compounds, such as nanoparticles coated by biomolecular corona^38^, drug-delivery vectors^39^, pathogens, sensors, etc, in analogy with the macropinosome example reported here. In general, this approach adds a dynamic dimension to standard co-localization studies, it allows for the real-time characterization of the intracellular evolution of the trafficking process, and offers a simplified experimental scheme (only one species labelled, one detection channel, and no additional data analysis to retrieve co-localization coefficients). From a technological point of view, this approach is highly flexible. For instance, being based on a standard spatiotemporal acquisition, it is fully compatible with virtually any kind of imaging modality, from camera-based (e.g. TIRF, SPIM) to scanning-based systems (e.g. confocal, STED-based imaging), from 1- to multi-photon excitation, etc., the only requisite being that the time resolution of the measurement can be properly tuned to the characteristic dynamics under study. In this regard, let us add that, as long as the available S/N ratio allows retrieving the characteristic MSD of the diffusing object from direct inversion of autocorrelation curve (as demonstrated by Shusterman and colleagues^40^), the fingerprint can be effectively extracted also from acquisitions in a single point in space. Lastly, the *i*MSD analysis can be combined with many others tools, either fluctuation-based (e.g. ICS, STICS, PLICS^41^) to increase the amount of information that can be extracted from standard imaging, or linked to the use of ‘intelligent’ dyes to probe selected intracellular parameters (e.g. pH, membrane order, etc.). Collectively, such implementations could transform the basic idea presented here in a flexible, multiplexed platform to address quantitatively the complex regulation of life at the subcellular level.

## Materials and Methods

### Cell culture and treatments

HeLa cells (CCL-2 ATCC) were cultured in Dulbecco’s modified eagle medium (DMEM) without phenol red (Gibco), supplemented with 10% fetal bovine serum (FBS, Gibco), 100 U/mL of penicillin, and 100 µg/mL of streptomycin in a humidified incubator at 37 °C and 5% CO_2_. Cells were seeded on 22-mm glass bottom dishes (WillCo Wells) and allowed to adhere overnight in a 37 °C and 5% CO_2_ cell culture incubator. CellLight Early Endosomes-GFP BacMam 2.0 (Invitrogen) and CellLight Late Endosomes-GFP BacMam 2.0 (Invitrogen) were used to mark early and late endosomes, respectively. Cells were transduced with CellLight reagents according to the manufacturer’s instructions. Briefly, cells were incubated with 40 µl of the CellLight solution with baculovirus in full growth medium overnight at 37 °C and 5% CO_2_. LysoTracker Red DND-99 (Invitrogen) stock solution was diluted to 60 nM final concentration in the growth medium. The medium from the dish was removed and pre-warmed (37 °C) probe-containing medium was added. Cells were incubated for 20 minutes and then observed. Transferrin from Human Serum conjugated to Alexa Fluor® 488 (Invitrogen) was used to label clathrin-mediated endocytosis. Transferrin conjugate was reconstituted in phosphate buffer saline (PBS) to obtain a 0.1 mM stock solution. Cells were incubated in a medium containing 20 nM of transferrin conjugate at 37 °C for 20 minutes then the medium was replaced. To investigate caveolae, cells were transfected with Caveolin-E^1^GFP by electroporation using Neon Transfection System 100 µL Kit (Invitrogen). Cells were trypsinized, pelleted, and resuspended in Resuspension Buffer R. DNA (15 μg) was added to 1 × 10^6^ cells in 220 μL buffer, followed by electroporation using Neon Transfection System (Invitrogen) operating at a voltage of 1005 V and width of 35 ms. The cells were then seeded and cultured in DMEM containing 10% FBS and supplements without antibiotics and used in experiments 24 h later. Fluorescein isothiocyanate-Dextran 70 kDa was used to label macropinosomes. Cells were washed three times with PBS, then the medium was substituted with Dextran-containing medium (1 mg/ml) and incubated at 37 °C for 30 minutes. At the end of the incubation period, cells were washed three times and the medium replaced. Microtubule depolimerization was induced by incubating the cells with 10 µM Nocodazole for 20 minutes and then washing with PBS before imaging.

### Live cell imaging

Confocal fluorescence image series were acquired with an Olympus FluoView FV1000 confocal microscope with a 60x NA 1.20 water immersion objective. All experiments were carried out at 37 °C and 5% CO_2_ using an incubation chamber enclosing the microscope stage and body. 488 nm Argon laser was used for excitation of early/late endosomes, caveolae, macropinosomes and clathrin-coated vesicles. The fluorescence emission was collected between 500 and 600 nm with the PMT detector in analog mode. 543 nm laser was used to excite Lysotracker. In this case, fluorescence emission was collected between 555 and 655 nm with the PMT detector in analog mode. The diameter of the detection pinhole was set to the size of 1 Airy. Sequential image series at 16 bits were collected at a fixed pixel size of 69 nm selecting a region of interest of 256 × 256 pixels and by varying the pixel dwell time from 0.5 to 2 or 4 µs per pixel depending on the characteristic diffusivity of the structure under study. The overall acquisition time varied from 30 seconds to 10 minutes, depending on the application.

### Image processing and data analysis

Both the *i*MSD processing of the acquired image-stacks and the subsequent data analysis were carried out with custom scripts working on MATLAB (MathWorks Inc., Natick, MA). In detail, we firstly computed by Fourier methods the spatiotemporal correlation function of the fluorescence intensity fluctuations g, which is defined as follows:

1 $$g(\xi ,\eta ,\tau )=\frac{\langle i(x,y,t)\cdot i(x+\xi ,y+\eta ,t+\tau )\rangle }{\langle i{(x,y,t)}^{2}\rangle }-1$$

where ξ and η are the distance between correlated pixels in the x and y directions, respectively, τ is the time lag, i(x, y, t) is the fluorescence intensity at point (x, y) and time t, and 〈…〉 indicates the average over spatial and time variables x, y and t^16^. g(ξ, η, τ) fit to standard Gaussian functions, i.e.

2 $$g(\xi ,\eta ,\tau )={g}_{0}+{g}_{1}(\tau )exp\{-\frac{{({\rm{\xi }}-{{\rm{v}}}_{{\rm{\xi }}}{\rm{\tau }})}^{2}+{({\rm{\eta }}-{{\rm{v}}}_{{\rm{\eta }}}{\rm{\tau }})}^{2}}{{{\rm{\sigma }}}^{2}({\rm{\tau }})}\}$$

where the numerator of the exponential term describes the net flux of particles along a specific direction in terms of average velocity, i.e. $\langle \vec{v}\rangle ={\vec{v}}_{\varphi }=({v}_{\xi },{v}_{\eta })$ and the variance σ^2^(τ) represents the mean square displacement of the ensemble as a function of the time lag. Thus, overall information about the intracellular dynamics has been obtained by the analysis of g(ξ, η, τ), without extracting and processing single particle tracks. As an instance, we carried out a straightforward categorization of motion by fitting σ^2^ to a power-law equation, i.e.

3 $${\sigma }^{2}(\tau )={\sigma }_{0}^{2}+\kappa \,{\tau }^{\alpha }$$

where σ_0_^2^ is an intercept value related to the average particle size and the waist of the point spread functions^16,18^ and α discriminates the dynamics as i) Brownian diffusion (α = 1), ii) super-diffusive motion (α > 1) and iii) sub-diffusion (α < 1). Furthermore, more accurate models allowed us to measure the involved dynamic parameters. More precisely, an anomalous diffusion with α < 1 can be regarded as a confined motion and the trend of σ^2^(τ) can be fitted to the following relationship^16^:

4 $${\sigma }^{2}(\tau )={\sigma }_{0}^{2}+4{D}_{M}\tau +\frac{{L}^{2}}{3}(1-\,e{\rm{xp}}\{-\frac{\tau }{{\tau }_{c}}\})\,$$

where L defines the linear size of the confinement area, τ_c_ is an index of how fast confinement occurs, D_M_ is the particle diffusivity at large time scale and represents 1/4 of the derivative of σ^2^ for τ → ∞. Similarly, the short-term diffusivity D_m_ is measured by the slope of σ^2^ for τ → 0 and reads ${D}_{m}={D}_{M}+{L}^{2}/(12{\tau }_{c})$ ^16^. On the other side, if α > 1, the trend of σ^2^(τ) is ascribable to the sum of a linear contribution due to Brownian diffusion and a parabolic term that describes a component of active transport along different directions on the focal plane^20^. In other words:

5 $${\sigma }^{2}(\tau )={\sigma }_{0}^{2}+4D\tau +{v}_{\sigma }^{2}{\tau }^{2}$$

where v_σ_^2^ represent the variance of particle velocity (i.e. ${v}_{\sigma }^{2}=\langle {(\vec{v}-\langle \vec{v}\rangle )}^{2}\rangle$) and D is the diffusion coefficient (which is the same both for short and long time scale). Therefore, we characterized the intracellular dynamics through Eqs 3–5. Finally, we point out the possibility to describe more complex dynamics, as for instance that of structures undergoing super-diffusive motion at a short time scale and confined diffusion at a larger time scale. To describe these systems, we propose the following generalization of the aforementioned models:

6 $${\sigma }^{2}(\tau )={\sigma }_{0}^{2}+4{D}_{M}\tau +\frac{{L}^{2}}{3}(1-\,e{\rm{xp}}\{-\frac{\tau }{{\tau }_{c}}\})+{v}_{\sigma }^{2}{\tau }^{2}\,{\rm{e}}{\rm{xp}}\{-\frac{\tau }{{\tau }_{v}}\}$$

where τ_v_ (τ_v_ < τ_c_) represents a characteristic time, below which the super-diffusive trend is dominant. Since the parabolic contribution decreases exponentially, at larger time delays it becomes negligible and the *i*MSD trend is determined by the confinement term. Worthy of note, this “global” model describes hybrid super/sub-diffusive behaviors within the employed correlation time window and preserves the physical meaning and the corresponding derivation of all the parameters, which are included in the previous descriptions. Finally, those models are included in Eq. 6 as particular cases, i.e. Eqs 4 and 5 can be regarded as limits of Eq. 6 for τ_v_ → 0 and τ_v_ → ∞, respectively.

## Electronic supplementary material

Supplementary Information
