# Supplementary material for: Dynamic fingerprinting of sub-cellular nanostructures by image mean square displacement analysis
Source: Sci Rep. 2017 Nov 1;7:14836. doi: 10.1038/s41598-017-13865-4 (PMC5665924; doi:10.1038/s41598-017-13865-4)
Supplement: Supplementary file 1 — Supplementary Information [file 41598_2017_13865_MOESM1_ESM.pdf]

Supplementary Information

Dynamic fingerprinting of sub-cellular nanostructures by image mean square displacement analysis

Luca Digiacomo[a,*], Francesca D'Autilia[b,*], William Durso[b], Paolo Maria Tentori[b], Giulio Caracciolo[a,†], Francesco Cardarelli[c,†]

[a] Department of Molecular Medicine, "La Sapienza" University of Rome, Italy.

[b] Center for Nanotechnology Innovation@NEST, Istituto Italiano di Tecnologia, Pisa, Italy.

[c] NEST - Scuola Normale Superiore, Istituto Nanoscienze - CNR (CNR-NANO), Pisa, Italy.

[*] These authors equally contributed

[†] To whom correspondence should be addressed: giulio.caracciolo@uniroma1.it, francesco.cardarelli@nano.cnr.it

Supplementary Figures

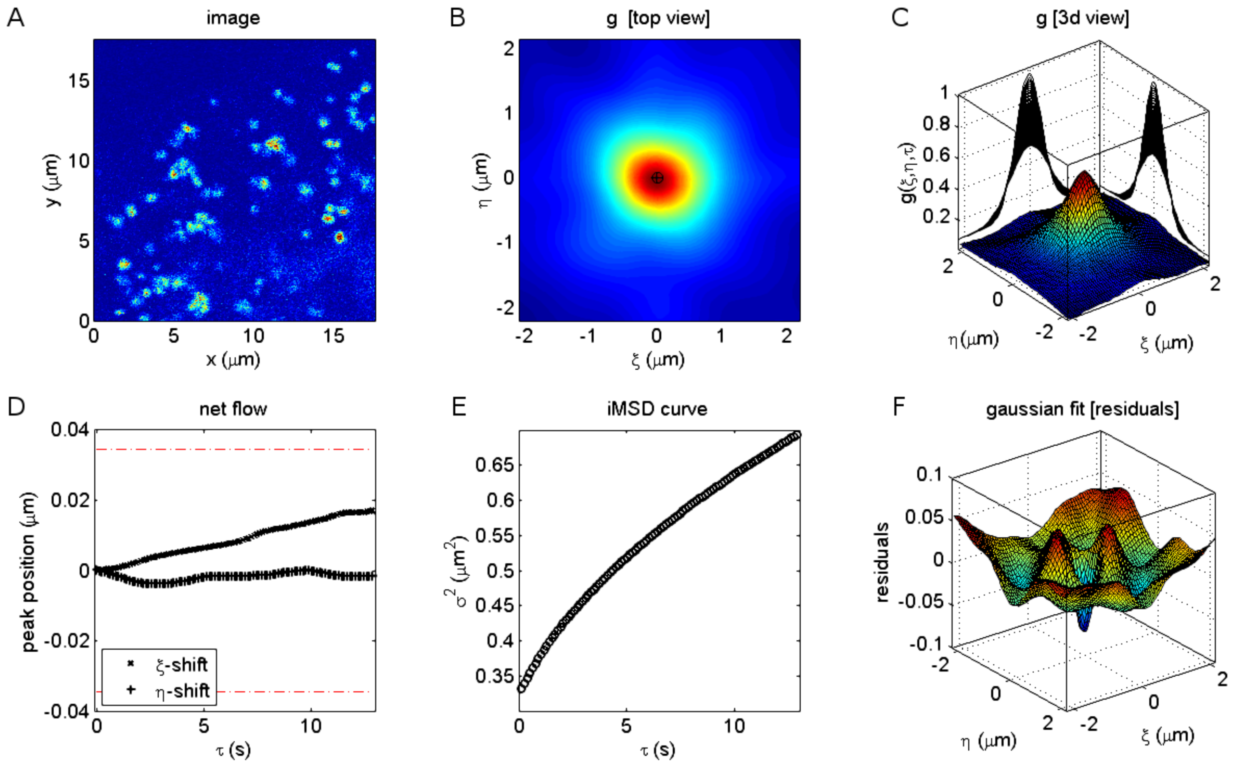

Supplemantary Figure 1 A) Representative image from the time-lapse B) Top view of the calculated spatiotemporal correlation function C) 3D view of the spatiotemporal correlation function, with projections D) Net movement of the peak of the spatiotemporal correlation function E) *i*MSD trace F) Gaussian fit residuals

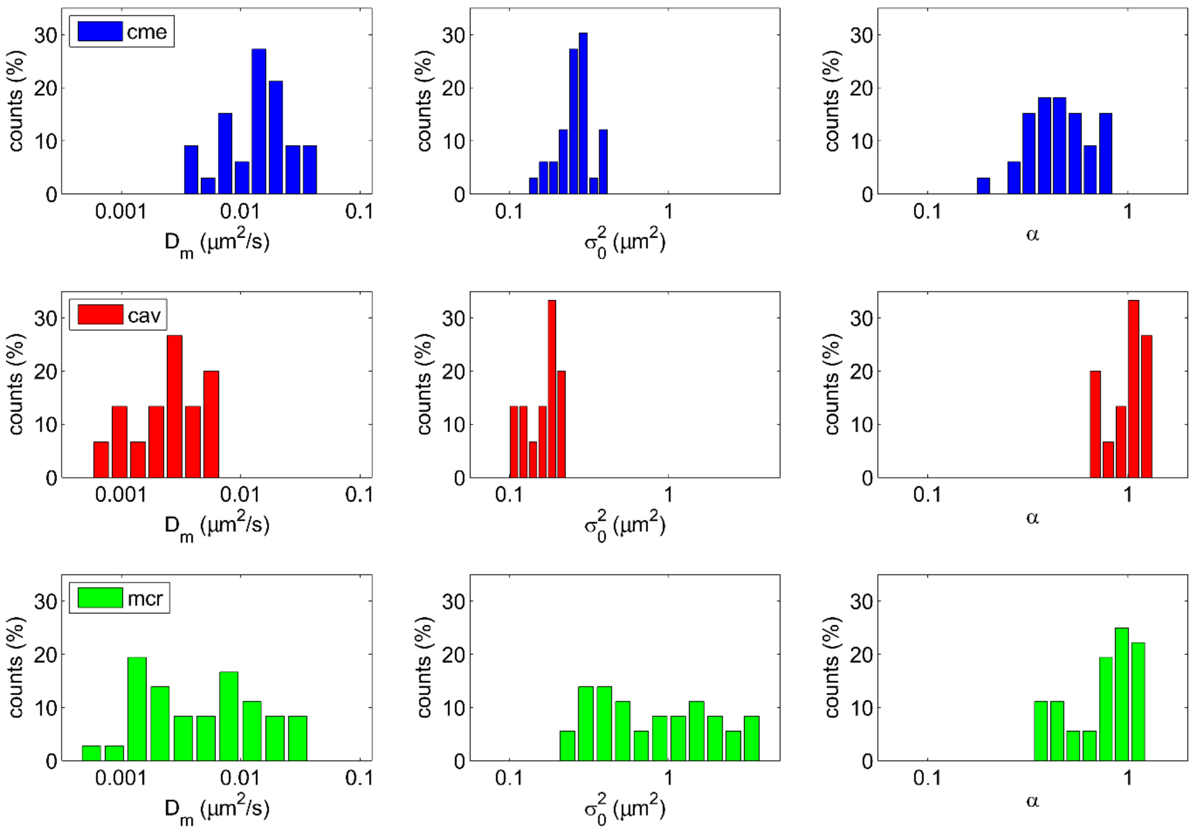

Supplemantary Figure 2 Distributions of D_m, σ_0^2, and α values for clathrin-mediated endocytosis (first row, blue), caveolae (second row, red), and early micropinocytosis (third row, green)

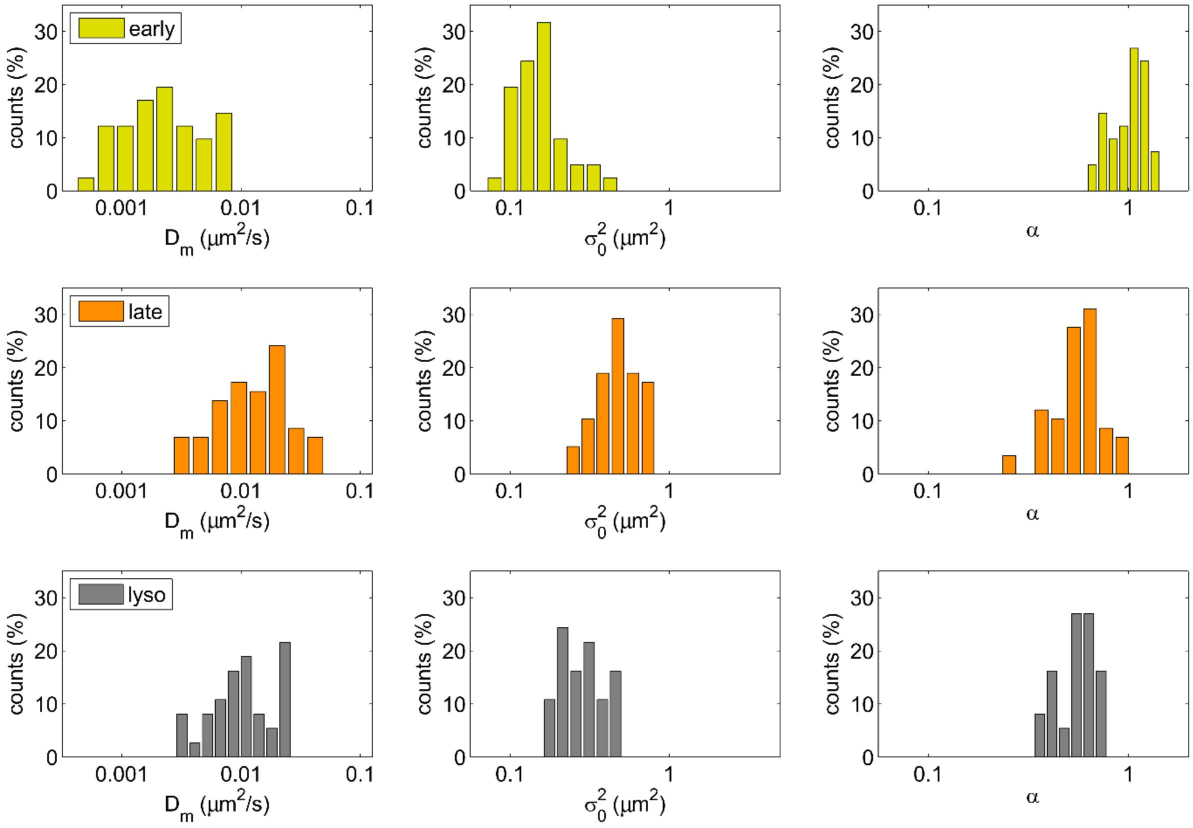

Supplemantary Figure 3 Distributions of D_m, σ_0^2, and α values for early endosomes (first row, dark yellow), late endosomes (second row, orange), and lysosomes (third row, grey)

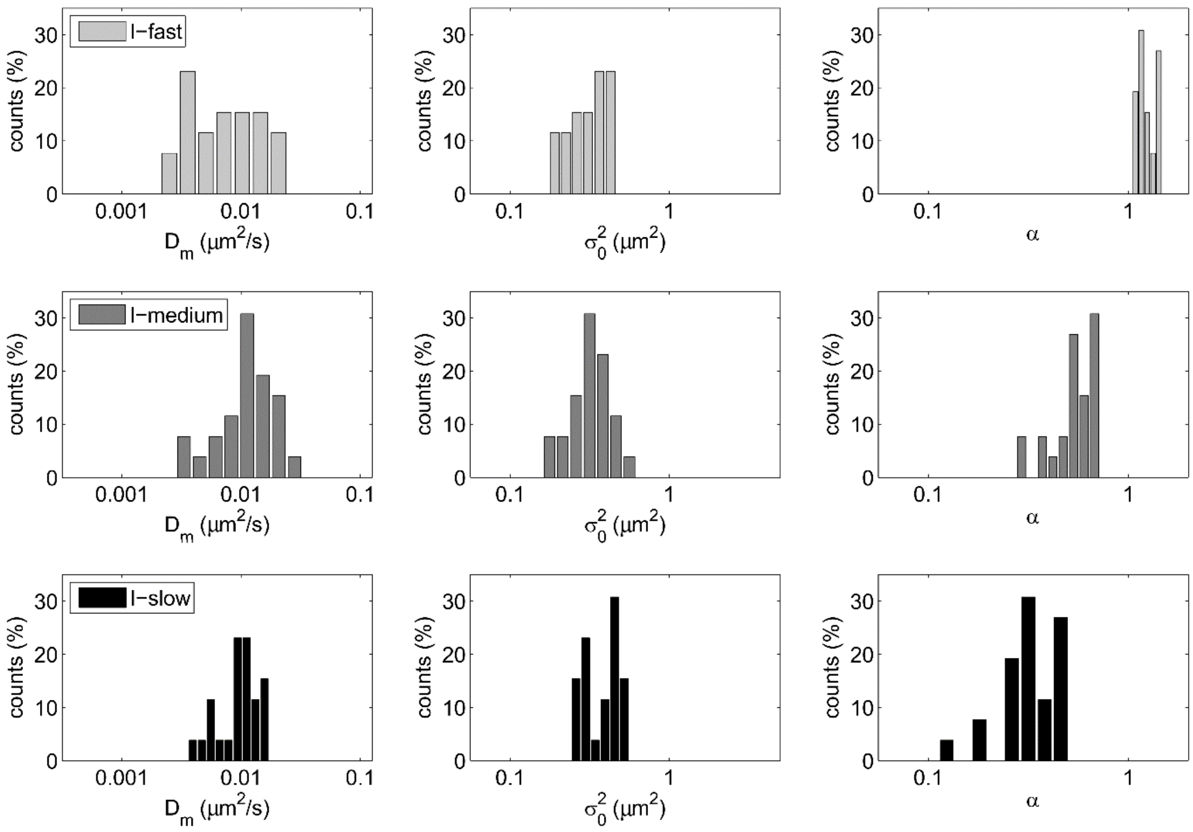

Supplemantary Figure 4 Distributions of D_m, σ_0^2, and α values for lysosomes in the 0-6 sec time window (first row, light grey), 0-12 sec time window (second row, dark grey), and 0-60 sec time window (third row, black)

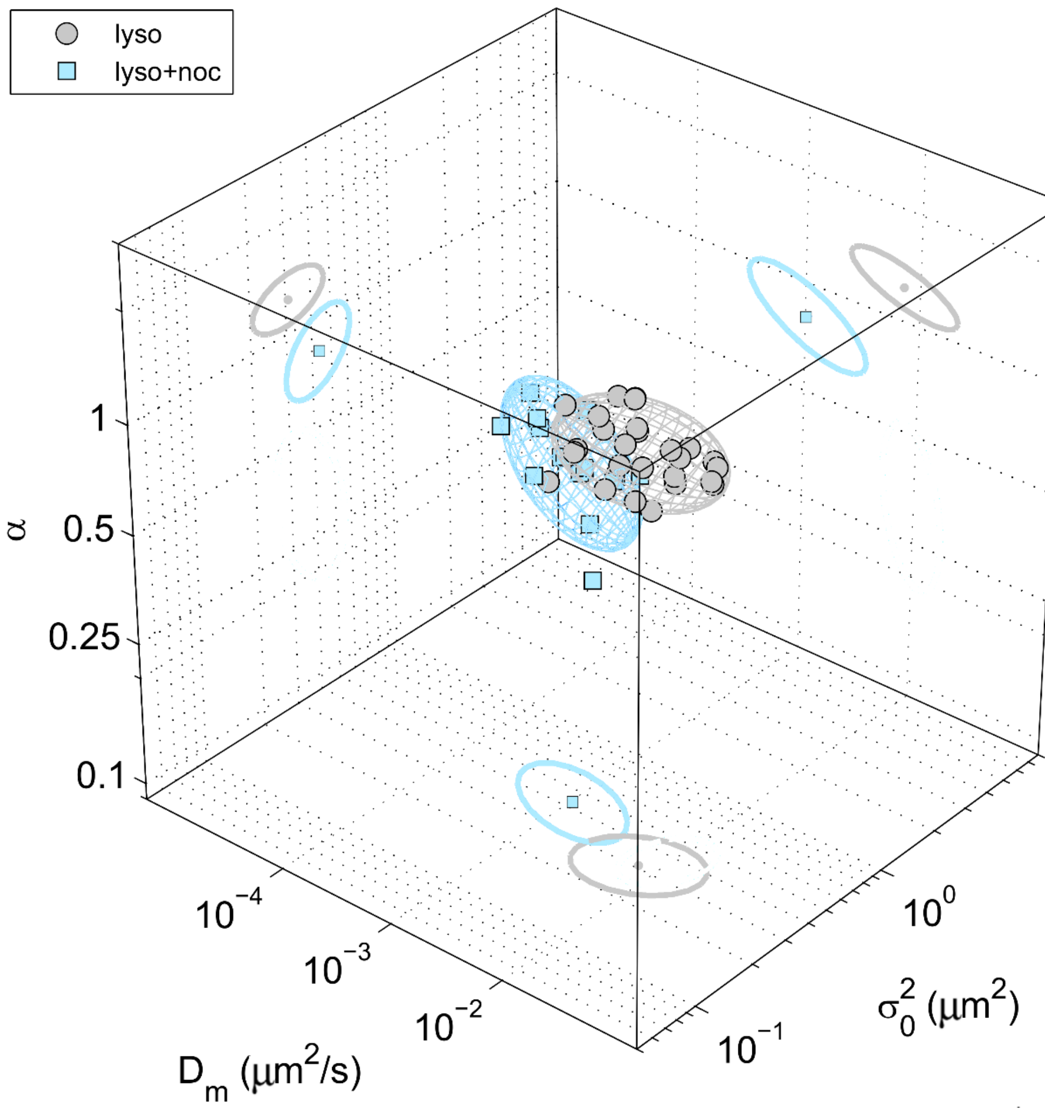

Supplemantary Figure 5 3D plot of experimental data points for lysosomes treated with Nocodazole (cyan), as compared to the control (grey).

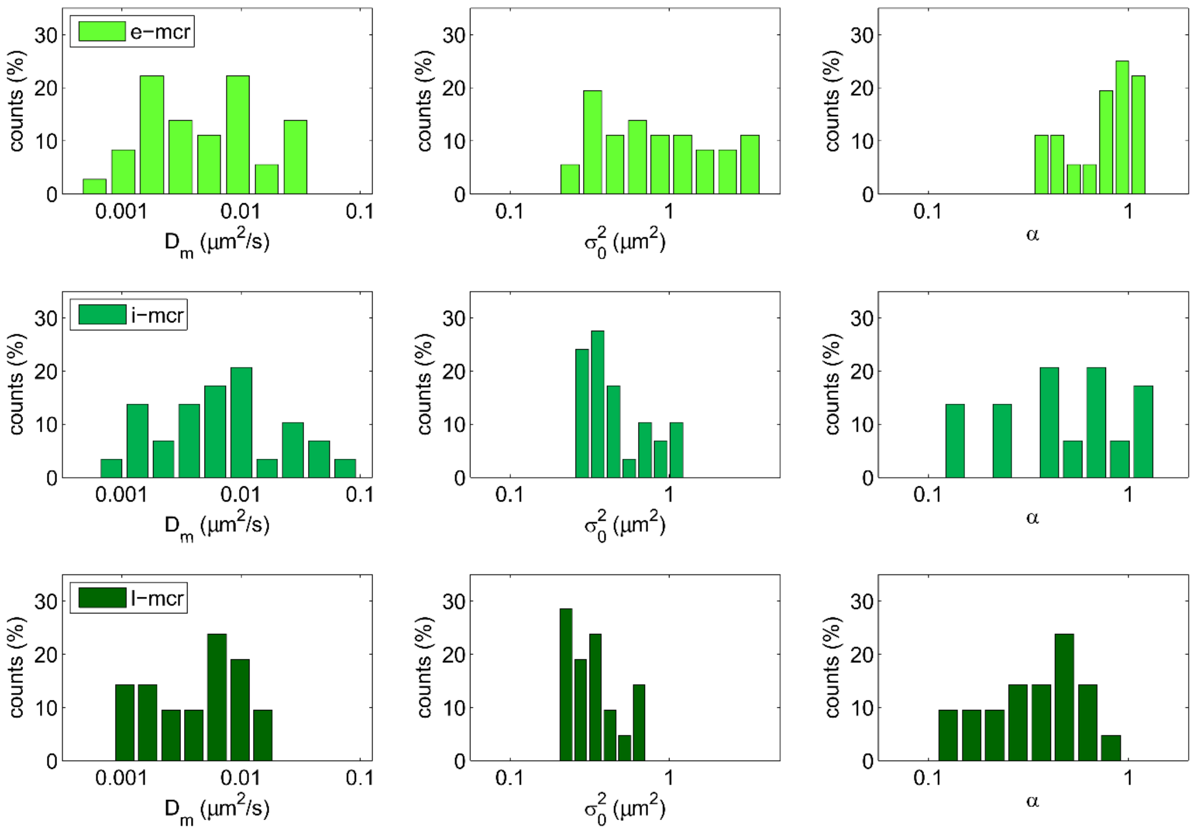

Supplemantary Figure 6 Distributions of D_m, σ_0^2, and α values for macropinosomes at different stages of trafficking, namely: early (30-70 min; first row, light green), intermediate (80-120 min; second row, green), and late (>120 min, third row, dark green)

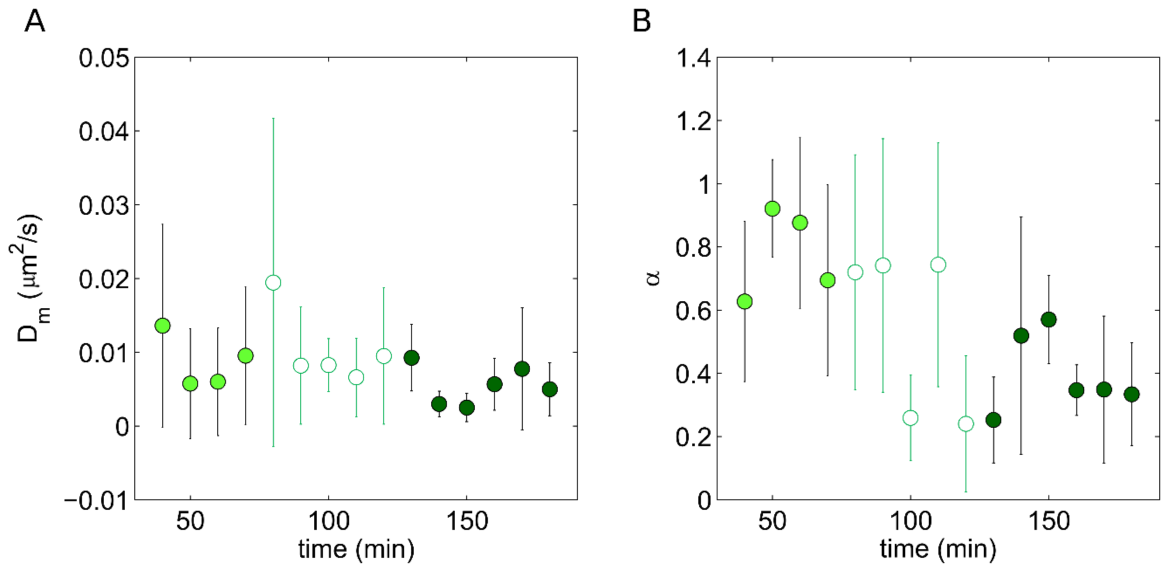

Supplementary Figure 7 A) Statistics on the average local diffusivity (D_m) of macropinosomes observed at the different time points. B) Statistics on the average anomalous coefficient (α) of macropinosomes observed at the different time points.
